# Autohydrolysis Application on Vine Shoots and Grape Stalks to Obtain Extracts Enriched in Xylo-Oligosaccharides and Phenolic Compounds

**DOI:** 10.3390/molecules28093760

**Published:** 2023-04-27

**Authors:** Graziana Difonzo, Marica Troilo, Michele Casiello, Lucia D’Accolti, Francesco Caponio

**Affiliations:** 1 Department of Soil, Plant and Food Sciences, University of Bari Aldo Moro, Via Amendola165, 70126 Bari, Italy; graziana.difonzo@uniba.it (G.D.); marica.troilo@uniba.it (M.T.); 2 Chemistry Department, University of Bari Aldo Moro, Via Orabona 4, 70125 Bari, Italy; michele.casiello@uniba.it (M.C.); lucia.daccolti@uniba.it (L.D.)

**Keywords:** xylo-oligosaccharides, prebiotics, antioxidant, by-products, winemaking

## Abstract

Agronomic practices and the winemaking process lead to the production of considerable quantities of waste and by-products. These are often considered waste with negative effects on environmental sustainability. However, vine shoots and grape stalks can be reused, representing a potential source of xylo-oligosaccharides and polyphenols. In this context, the purpose of this work was to obtain enriched extracts using three different autohydrolysis treatments with (i) H_2_O, (ii) H_2_O:EtOH, and (iii) H_2_O:Amberlyst. The obtained extracts were characterized by their xylo-oligosaccharide and polyphenol profiles using LC-MS techniques. The use of ethanol during autohydrolysis allowed for greater extraction of xylan-class compounds, especially in vine shoot samples, while an increase in antioxidant activity (128.04 and 425.66 µmol TE/g for ABTS and DPPH, respectively) and in total phenol content (90.92 mg GAE/g) was obtained for grape stalks.

## 1. Introduction

Xylo-oligosaccharides (XOS) are sugar oligomers composed of two to ten units of xylose, which are bound by β(1→4)-xylosidic linkages [1,2,3,4] and which form branched structures when linked to different substituents, such as acetyl groups, glucuronic acids, and arabinose and galactose residues [4,5,6]. Mainly, XOS are produced by hydrolysis of xylan, the main constituent of cellulose polysaccharides present in plant cell walls [2,5,7]. Depending on the source of xylan and the production process, these compounds vary in terms of the degree of polymerization (DP), structure, and type of bonds present [6,8]. The global XOS market is expanding, and a compound annual growth rate (CAGR) of 1.5% is expected to reach a total of 33 million dollars during 2023–2029 [9].

From the nutritional point of view, XOS are recognized for their high prebiotic power. Prebiotics are digestible food ingredients with related benefits in the host as they promote selective growth of beneficial bacteria in the colon [6].

The beneficial effects of these compounds are related to (i) the growth of prebiotic bacteria in the intestinal tract, (ii) the prevention of diabetes and reduction of glycemic index and blood cholesterol, (iii) the stimulation of the immune system, (iv) the prevention of inflammation of the colon, and (v) the improvement of the absorption of minerals in the intestine [5,10]. The prebiotic activity of XOS is linked to their ability to induce growth of prebiotic microorganisms, including *Bifidobacterium* and *Lactobacillus*. Many studies show how the presence of XOS determines an increase in the cell density of *bifidobacteria* when these sugars are used as a carbon source. In addition, the ability of these molecules to reduce the number of *Clostridium* has been shown, as well as their ability to reduce the concentration of secondary bile acids, compounds potentially related to cancer promotion and negative impacts on the colon [8].

Compared with the other prebiotic compounds, the XOS have high-temperature stability of up to 100 °C and a wide pH range (from 2.5 to 8), and therefore are easily usable in food applications [11,12]. In fact, XOS can be used in fruit juices with low pH or in acidic foods and carbonated drinks [6]. In addition, XOS are often used as emulsifying agents, stabilizers, and substitutes for fats and sugar in food, zootechnical, and cosmetic sectors [8,13,14,15].

It is important to highlight that though these interesting compounds are naturally contained in different types of foods, such as fruits and vegetables [1,6], they are not present in sufficient quantities to exert the prebiotic effect [16]. For this reason, it is necessary to supplement foods with XOS recovered from natural sources, such as agricultural biomass or waste and by-products of agrifood chains [17].

Vine shoots and grape stalks—with an annual estimated production of 1–2 tons per hectare and 7% *w*/*w* of grape total weight [18], respectively—could be considered a potential source of XOS; in fact, they consist mainly of three fractions—cellulose, hemicellulose, and lignin [19]—and can therefore be used for lignocellulosic fractionation processes in order to break down polymers and promote the XOS production [20].

The growing focus on environmental sustainability has led researchers to develop alternative and innovative methods for food-grade XOS recovery [21]. Among these, enzymatic and hydrothermal hydrolysis were most investigated [6,22]. However, using only water treatment limits the optimal production of XOS. To tackle this problem, ethanol [23] could be the right compromise between increasing the extraction yield of XOS and the need to use *green* solvents [24]. Moreover, another alternative could be the use of Amberlyst, an insoluble macroporous ionic exchange resin that can facilitate hydrolysis phases [25]. The particularity of this catalyst lies in the presence of active acid sites linked to a copolymer and characterized by sulphonic groups derived from sulphuric acid. In addition, the swelling properties induced by the solvent (water), make acidic sites accessible, facilitating the hydrolysis phases for the release of H^+^.

With regards to the oenological lignocellulosic materials, the autohydrolysis treatments were widely applied previously, testing different combinations of temperature and/or time [20,26,27,28]. However, for the applied experimental conditions, a post-hydrolysis treatment with alkaline and/or acid solutions was necessary for a total recovery of XOS.

In addition to the possibility to extract XOS from grape stalks and vine shoots, these are a source of several bioactive compounds, such as polyphenols [18,19].

In this framework, the present study aimed to evaluate the extraction yield in XOS applying three different green treatments of autohydrolysis: (i) H_2_O, (ii) H_2_O:EtOH (70:30 *v*/*v*), and (iii) H_2_O:Amberlyst (95:5 *w*/*w*). An innovative identification and quantification method of the extracted sugars by the LCMS-IT-TOF system, not previously considered for these constituents, was used. This technique is well known to provide more detailed information on the structure of these compounds [29]. In addition, the obtained extracts were evaluated for their phenolic profile and antioxidant activity.

## 2. Results and Discussion

### 2.1. Qualitative Analysis of Extracts

Analysis of complex mixtures of sugars is a difficult procedure [30] as it requires the ability to discriminate compounds (especially in the case of monosaccharides) that sometimes have the same molecular formula and identical moieties. Therefore, the analysis of the mass spectra is notably difficult and requires appropriate derivatizations to be effective [31].

Considering that in this work the focus is not to obtain a fine characterization of the compounds but to verify the presence of saccharides useful as additives and to simplify the characterization procedures, LC-MS techniques were used to analyze the samples without any pretreatment either for qualitative or quantitative identification. In particular, the identification of the compounds was conducted through the analysis of the exact masses and the analysis of isotopic and fragmentation patterns.

The analysis of the exact masses and isotopic patterns was conducted with the SHIMADZU software package that includes Formula Predict and Accurate Mass Calculator with which the main components of the extracted matrix were identified.

**Figure 1:**
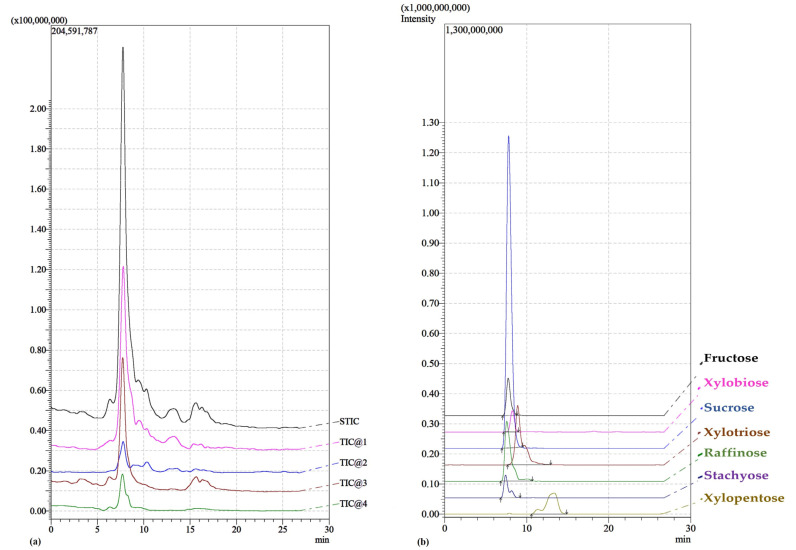
(**a**) An example of a chromatogram plot (TIC, total ion current) of the mixture. The five signals represent the following starting from the top: Sum TIC, Positive TIC, Positive Fragment TIC, Negative TIC, and Negative Fragment TIC. (**b**) Single trace of the various sugars.

Figure 1 shows a typical chromatogram (TIC, total ion current), obtained from the analysis of extracts with water from the grape stalks. The chromatograms of the other matrices appear to be similar.

As can be seen, the C18 column was not able to perform a perfect separation (Figure 1a); nevertheless, through the extrapolation of the chromatograms of the single ions, it was possible to obtain the traces of the compounds studied. In Figure 1b, it is possible to see how the peaks of the single sugars, emerge from the global signal. This operation allows the qualitative identification and, also, measuring of the areas of signals for the purpose of quantification.

Table 1 lists the species identified with the respective *m*/*z* values compared with the theoretical ones and the difference in ppm.

As expected, the formation of the adduct with the Na^+^ cation is preponderant among all sugars [32]. All measured *m*/*z* values have a deviation of less than 15 ppm compared with the theoretical ones and in some cases less than 2 ppm allowing us to have a solid certainty on the attributions of the various signals.

**Figure 2:**
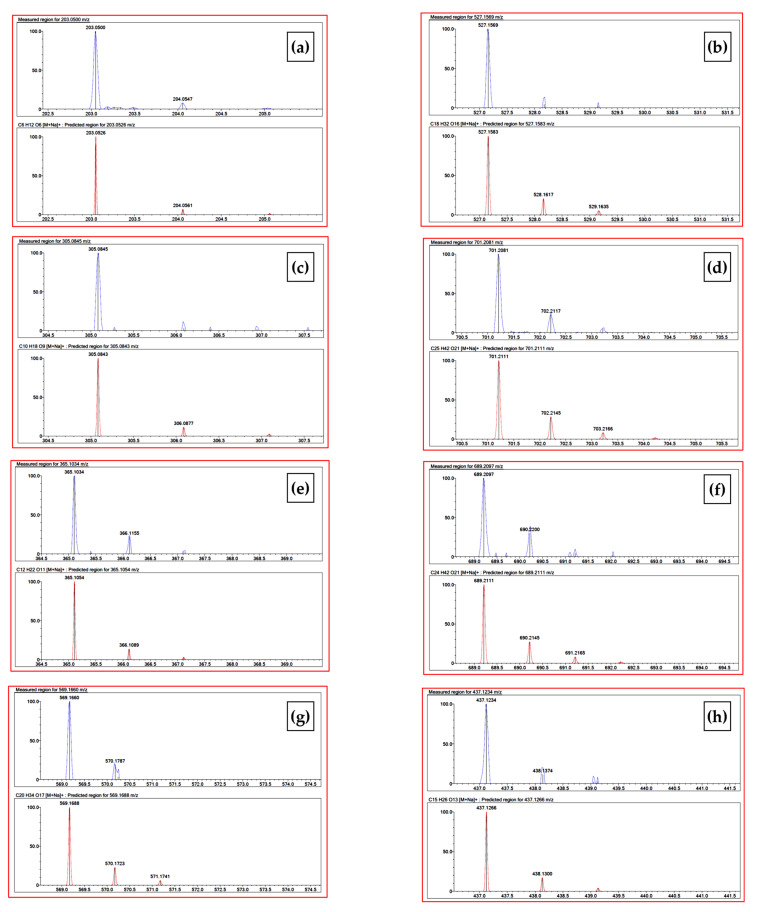
Measured isotopic pattern (blue) compared with theoretical one (red): (**a**) fructose; (**b**) raffinose; (**c**) xylobiose; (**d**) xylopentose; (**e**) sucrose; (**f**) stachyose; (**g**) xylotetraose; (**h**) xylotriose.

Further verification was obtained through the analysis of the isotopic patterns. In this case, the software analysis provides us with additional confirmation of the origin of the signals. Figure 2 shows the experimental mass signals with relative isotopic patterns (blue traces), compared with the theoretical ones (red traces) suggested by the software. It is easy to see a high degree of overlap.

### 2.2. Quantitative Analysis of Extracts

The quantitative analysis was carried out following the identification of various species of sugar identified in the extracts through the construction of appropriate calibration curves, by preparing standard solutions of the sugars identified. The results are reported in Table 2 and Table 3.

Table 2 shows the data concerning the mono- and oligosaccharides composition of the extracts of vine shoots (VS), obtained following three different extraction methods.

As expected, xylo-oligosaccharides have been the main components in all extraction methods. In particular, the presence of ethanol seems to have a positive influence on the extraction of oligosaccharides such as xylotetraose and xylopentose, present in quantities of 21.35 and 45.98 g/100g. This could be due to poor solubilization of hemicellulose in water and partly in an organic solvent. For this reason, the use of a mixture of H_2_O-EtOH could perform a polysaccharide removal action [33]. Therefore, as highlighted in Table 2, the concentration of tri- and tetrasaccharides, as well as pentasaccharides, increased when 30% ethanol was used. The organic solvent, in fact, induced a high dissolution and release of these compounds.

Autohydrolysis treatments with water and with the addition of Amberlyst, on the other hand, increased the concentration of raffinose and stachyose. In fact, their hydrolysis was not high with the use of ethanol. As reported by Hu et al. [34], this may be due to a steric hindrance to accessing the catalytic sites owing to the big molecular size of these compounds. Amberlyst, releasing H^+^ ions from sulfonic groups present on resin, allows both to promote the hydrolysis of oligosaccharides in monosaccharides and disaccharides and to operate partial hydrolysis of the cellulose providing their release in the extracts. This is a result of the swelling of the resin, which allows it to obtain a high concentration of H+ able to hydrolyze the cellulose and release polysaccharides.

Contrary to VS, the positive effect of ethanol on the extraction of xylo-oligosaccharides was not observed in the grape stalks (GS) extracts. As shown in Table 3, xylotetraose and xylopentose were more concentrated in the aqueous extract obtained with the use of H_2_O and H_2_O:Amberlyst than the solution of H_2_O:EtOH. The latter, instead, has contributed to the increase in the sucrose content, and particularly in the fructose content, as was also observed in VS samples.

Moreover, the two matrices showed a similar trend in the extraction of oligosaccharides such as raffinose and stachyose. Ethanol has significantly reduced their concentration compared with the other two treatments used.

It is well known that the raffinose family oligosaccharides (raffinose, stachyose, and verbascose) belong to the category of antinutritional compounds. Their presence has been identified not only in legumes but also in storage organs, such as roots, tubers, and plant woody organs [35]. They are not absorbed and hydrolyzed in the upper gastrointestinal tract, and, thus, they get accumulated in the large intestine of the human digestive system. This is also related to the lack of the enzyme α-galactosidase capable of hydrolyzing the galactosidic bond α -D- (1,6) in the small intestine [36]. As a consequence, microbial fermentation by colon bacteria develops at a certain level of these oligosaccharides, leading to the formation of hydrogen, methane, and CO_2_, responsible for flatulence [35,37,38]. In addition, these gases cause abdominal discomfort, cramps, diarrhea, and nausea [35,38]. At the same time, recent studies highlighted the benefit of these compounds for human health, linked both to the possibility of conversion into prebiotic molecules, promoting the growth of *Bifidobacteria* and *Lactobacilli* at the expense of bacteria harmful to the colon, and to different anti-allergic, antidiabetic and anti-obesity properties [35,39].

Given these established effects, it is necessary to explore the appropriate dose needed to achieve positive effects without side effects. In this study, the use of H_2_O:EtOH solution could represent a good compromise between high extraction of prebiotic compounds (xylo-oligosaccharides) and minor hydrolysis and release of oligosaccharides such as raffinose and stachyose.

XOS seem to be more concentrated in the VS extracts, in particular following the use of organic solutions; GS, on the other hand, seems to be an excellent source of monosaccharides, such as fructose, probably because of the greater contact with the grape juice that impregnate the grape stalks during the crushing and destemming phases [40,41].

### 2.3. Phenolic Compounds Characterization

Recently, the interest in recovering phenols as added-value antioxidant compounds has been increasing, which is why total phenol content and antioxidant capacity were also evaluated in the extracts obtained from vine shoots and grape stalks by autohydrolysis.

In fact, different studies reported that these matrices are a food source of antioxidant compounds, such as polyphenols [42,43,44,45,46,47,48]. Table 4 shows the antioxidant activity and the total phenol content of the extracts from VS and GS; the extracts obtained with an aqueous solution showed the highest ABTS and DPPH values and total polyphenols content (65.67 and 250.14 µmol TE/g, and 58.03 mg GAE/g respectively). Otherwise, the extract from GS obtained using H_2_O-EtOH was the richest in phenolic compounds (90.92 mg GAE/g) and showed the greatest values in antioxidant activity (128.04 and 425.66 µmol TE/g), as also found by Jiménez-Moreno et al. [44].

As highlighted, the polyphenols extraction from two plant matrices was influenced by the type of solvent used. These differences could be related to the matrix effect and probably to different chemical compositions. The solubility and the extraction efficiency of polyphenols in different solvents are influenced by their chemical nature and different polarity, as well as by the presence of interfering substances, which vary depending on the matrix analyzed [49,50]. For example, Vural et al. [50] and Bhebhe et al. [51] showed higher extraction of phenolic compounds in tea samples due to the use of hydroalcoholic solutions compared with aqueous solvent only. The results of the antioxidant activity reflect the total phenolic content, highlighting that in this case, the polyphenols are the molecules that most contributed to the antioxidant power of the extracts.

Overall, the different extraction efficiency could be due to the structure, porosity, and different chemical composition of the sample, which changes the penetration of the solvent and, consequently, the amount of polyphenols found.

To investigate the main phenolic compounds in the extracts, an LC-ESI-MS/MS method was applied in this study. Negative ion mode was selected for the generation of spectra because of its better sensitivity for most of the phenolic compounds investigated.

The profiles showed small variations in relation to different extraction solvents and among vine shoots and grape stalks. The main compounds were identified as reported in Table 5. The phenolic compounds were identified on the basis of retention times, MS/MS fragmentation, and the literature data as a comparison [52,53,54,55,56,57,58,59,60,61,62,63,64].

**Figure 3:**
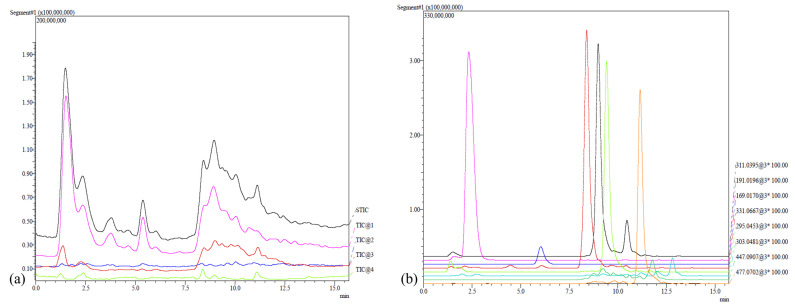
(**a**) An example of a chromatogram plot (TIC, total ion current). The five signals represent the following starting from the top: Sum TIC, Positive TIC, Positive Fragment TIC, Negative TIC, and Negative Fragment TIC. (**b**) Single trace of the various phenolic compounds.

Figure 3 shows a chromatogram (TIC, total ion current), obtained from the analysis of all extracts.

The detected phenolic compounds were gallic acid, monogalloyl glucose, (epi)gallocatechin, caftaric acid, coutaric acid, quercetin-3-O-glucuronide, Kaempferol-3-O-glucoside, and taxifolin.

## 3. Materials and Methods

### 3.1. Chemicals and Reagents

Methanol (HPLC grade) and ethanol absolute anhydrous were purchased from Carlo Erba (Milan, Italy); sodium carbonate, water (LC-MS, Ultra Chromasolv, Honeywell, Seelze, Germany), and acetonitrile (LC-MS Chromasolv > 99.9%) from Honeywell (Seelze, Germany). Formic acid (99% LC-MS grade) was purchased from VWR Chemicals (Radnor, PA, USA). Amberlyst 15 ion-exchange resin, Folin–Ciocalteu reagent, ABTS (2,20-azino-bis (3-ethylbenzothiazoline-6-sulphonic acid) diammonium salt) and DPPH (2,2-diphenyl-1-picrylhydrazyl) were purchased from Sigma-Aldrich (St. Louis, USA). Standard of D-(+)-saccharose molecular biology grade was purchased from AppliChem GmbH (Darmstadt, Germany); standards of D-(+)-raffinose and stachyose tetrahydrate were purchased from Supelco analytical (Darmstadt, Germany); standard of L-(−)-xylose and D-(−)-fructose were purchased from Sigma (St. Louis, MO, USA).

### 3.2. Vine Shoots and Grape Stalks Preparation

Vine shoots and grape stalks (*Vitis vinifera* L., cultivar ‘Bombino Nero’) were collected at a winery in Corato (Bari, Italy), following pruning and destemming phases, respectively, in March and September 2021. Vine shoots were cut in a hammer crusher and then dried at 120 °C for 1 h in a ventilated oven (Argol Lab-TCF120) to obtain a moisture content of 5%, measured with a thermobalance (Radwag Mac 110/NP, Radom, Poland), while grape stalks were dried at 120 °C for 45 min to obtain a moisture content of 3–4%, as described by Troilo et al. [65]. Subsequently, both biomasses were ground in a mill (Vercella, ETA model, Turin, Italy) and then sieved with a 425 µm stainless steel sieve.

### 3.3. Hydrothermal Treatments of Vine Shoots and Grape Stalks

**Figure 4:**
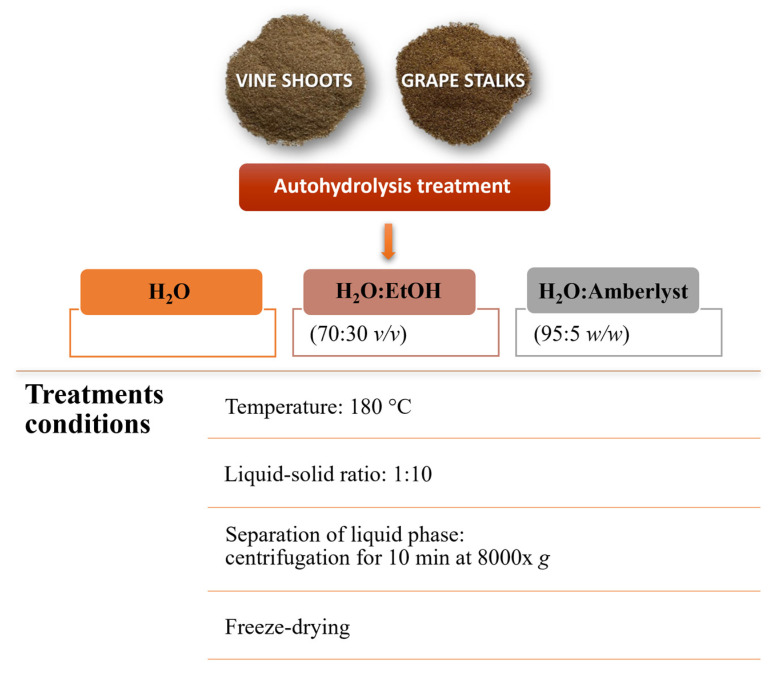
Scheme of autohydrolysis treatment.

In order to obtain extracts rich in XOS, vine shoots and grape stalks have been subjected to different hydrothermal treatments, as described by Dávila et al. [20] and Gullon et al. [27] with some modifications. Briefly, green solvents, such as water and ethanol, were used, and three methods of autohydrolysis were tested: (i) extraction with H_2_O; (ii) extraction with H_2_O:EtOH (70:30 *v*/*v*); and (iii) extraction with H_2_O:Amberlyst (95:5 *w*/*w*). All treatments were carried out at 180 °C for 2 h in a stainless-steel reactor at the liquid-solid ratio of 1:10. After cooling, the liquid fraction was separated from the solid phase by centrifugation for 10 min at 8000× *g* and then lyophilized. The extraction was carried out in duplicate (Figure 4).

### 3.4. Characterization of the Autohydrolysis Liquors

The extracts deriving from the autohydrolysis treatment were analyzed by dissolving the dried extract in HPLC-grade water in a 1:33 ratio. The analyses were carried out using an LCMS-IT-TOF system (Shimadzu, Tokyo, Japan), consisting of a binary pump (NexeraXR, LC-20ADxr), autosampler (NexeraXR, SIL-20ADxr), and a detector (SPD-M20A), as described by Li et al. [66] and De Leo et al. [29] with some modifications.

For the separation of the mixtures, a C18 column (150 × 4.6 mm) with packing particles of 5 µm (Supelco), installed in a thermostated oven at a temperature of 40 °C, was used. The mobile phase was composed of a solution of H_2_O/formic acid (99.9/0.1 *v*/*v*) (A) and acetonitrile/methanol (4:3 *v*/*v*) containing 0.1% of formic acid (B). The gradient of phase B was set as follows: 0–16 min 2%; 16–20 min 20%; 20–30 min 60%; 30–45 min 100%; 45–70 min 2%, total flow set to 0.25 mL/min. The total run time was 70 min, with an injection volume set to 5 µL.

The ESI interface was set in the positive and negative mode, CDL (curved desolvation line) and heat block temperature were set to 240 °C, the mass screening range was 100 to 2000 *m*/*z*, the detector voltage was set to 1.7 kV, and the nebulizing gas, consisting of nitrogen, was set with a flow of 1.5 L/min. The software used for data acquisition and processing was the LCMS solution (V3.80.410, Shimadzu). The calibration curves, statistical calculations, and qualitative characterization were processed using the Shimadzu LabSolutions Lite V5.82 software package and the Minitab^®^ 21.3.1 (64-bit) in combination.

Calibration curves were prepared for quantitative characterization. For each sugar’s standard, up to 4 decreasing concentration solutions were prepared by diluting a mother solution with HPLC-grade water. Each solution was analyzed up to 4 times. LOD and LOQ were calculated for fructose (0.0025 and 0.0082 mmol/L), sucrose (0.0128 and 0.0425 mmol/L), xylose (0.0058 and 0.0196 mmol/L), raffinose (0.0008 and 0.0029 mmol/L), and stachyose (0.0027 and 0.009 mmol/L) calibration curves.

All the chromatogram areas, the calibration curves, and the respective parameters of the various standard species were automatically calculated by inputting in the software the following parameters: (i) signal amplitude 10 s; (ii) slope 10,000 uV/min; (iii) minimum area of 1000 units; (iv) retention time specific to the species being analyzed; (v) *m*/*z* value of the species under examination; and (vi) smoothing normal mode, 5 iterations.

The software calculations used linear regression, an equation of the type Y = mX + b, and statistical parameters, such as standard deviations.

The separation of phenolic compounds was performed with an Hypersil Q C18 column (1.9 µm particle size, 2.1 mm × 100 mm length, Thermo Fischer Scientific), maintained at 30 °C, using a mobile phase consisting of (A) water/formic acid (99.9:0.1, *v*/*v*) and (B) acetonitrile/formic acid (99.9:0.1 *v*/*v*), at the constant flow rate of 0.3 mL/min. The gradient program of solvent B was as follows: 0–20 min from 2% to 70%; 20–24 min isocratic at 70%, 24–24.3 from 70% to 2%, 24.3–33.7 min an isocratic at 2%. The MS parameter conditions were taken by Makhlouf et al. [67] with some modifications: capillary temperature 320 °C; source heater temperature 280 °C; nebulizer gas N_2_; sheath gas flow 33 psi; auxiliary gas flow 5 arbitrary units; and S-Lens RF Level 60%. Data were acquired in negative ionization mode. Samples were analyzed with a full scan method from 100 to 1500 *m*/*z* and a data-dependent experiment to collect MS^2^ data.

The samples were filtered using syringe filters in RC by 0.22 µm before injection into the equipment. All data were acquired and processed using LCMS solution (V3.80.410, Shimadzu). The injection volume was 5 µL. Tentative identification of compounds was performed using mass spectra (MS^2^) in the literature [52,53,54,55,56,57,58,59,60,61,62,63,64].

### 3.5. Determination and Quantification of Phenolic Profile

The total phenol content (TPC) was determined using the Folin–Ciocalteu method according to Difonzo et al. [68]. In particular, 20 µL of filtered extracts and 100 µL of Folin–Ciocalteu reagent was added to 980 µL of deionized water. After 3 min, 800 µL of 7.5% Na_2_CO_3_ was added, and then incubated at room temperature for 60 min. The absorbance was read at 720 nm using a Cary 60 spectrophotometer (Cernusco, Milan, Italy), and the results were expressed as mg of gallic acid equivalents (GAE)/g of sample. Each sample was analyzed in triplicate.

### 3.6. Antioxidant Activity Evaluation

The extracts were analyzed for the evaluation of antioxidant activity with DPPH and ABTS assays, as described by Difonzo et al. [69]. The DPPH assay was carried out by preparing a solution of DPPH 0.08 mM in ethanol. Then, in cuvettes for spectrophotometry, 50 µL of the sample was added to 950 µL of DPPH solution. After 30 min of incubation, the absorbance was read at 517 nm using a Cary 60 spectrophotometer. However, for the ABTS assay, an ABTS˙^+^ radical was generated by a reaction with potassium persulfate (K_2_S_2_O_8_), adding 25 mL of ABTS (7 mM in H_2_O) to 800 µL of K_2_S_2_O_8_ and incubated in the dark for 16 h. The reaction for evaluating the antioxidant activity was carried out in cuvettes for spectrophotometry, with 50 µL of each sample and 950 µL of ABTS˙^+^ solution. After 8 min, the absorbance was read at 734 nm. The results were expressed in µmol Trolox equivalents (TE)/g of samples. Each sample was analyzed in triplicate.

### 3.7. Antioxidant Activity Evaluation

Analysis of variance (ANOVA) and Tukey test were carried out on the experimental data by Minitab Statistical Software V19 (Minitab Inc., State College, PA, USA). The assumptions in terms of homogeneity of variance, independent residuals, and normal distribution of residuals were guaranteed, and differences were considered statistically significant at *p* < 0.05.

## 4. Conclusions

In recent years, the attention paid to waste in the agrifood supply chains is constantly growing because it represents an important environmental issue. The wine sector is characterized by both residues of processing phases and those of pruning, such as grape stalks and vine shoots, which are rich in bioactive compounds, especially xylo-oligosaccharides, and polyphenols, and have nutraceutical properties, with a potential for wide use in the production of additives, ingredients, and functional products.

Three autohydrolysis extraction methods were applied to extract functional compounds from the grape stalks and vine shoots. The extracts were characterized for XOS and the main phenolic compounds using LC-MS techniques. In vine shoots, ethanol had a positive impact on the dissolution of xylo-oligosaccharides consisting of four and five units of xylose. On the other hand, in the grape stalks, similar results were found using aqueous solutions. In both matrices, the use of the hydroalcoholic solution allowed us to obtain a decrease in the concentration of oligosaccharides, such as stachyose and raffinose, known antinutritional compounds. On the other hand, the use of an H_2_O:EtOH mixture increased the extraction of antioxidant compounds, especially in grape stalk samples.

## Figures and Tables

**Table 1:** The exact mass of identified sugars.

Sugars	Molecular Formula	Exact Mass (Molecular Ion)	Predicted Mass (Na Adduct)	Measured Mass	Diff PPM
*Fructose*	C_6_H_12_O_6_	180.0634	203.0526	203.0501	12.3121
*Stachyose*	C_24_H_42_O_21_	666.2219	689.2111	689.2101	1.4509
*Xylobiose*	C_10_H_18_O_9_	282.0951	305.0843	305.0848	1.6389
*Raffinose*	C_18_H_32_O_16_	504.1690	527.1583	527.1541	7.9673
*Sucrose*	C_12_H_22_O_11_	342.1162	365.1054	365.1083	7.9429
*Xylotriose*	C_15_H_26_O_13_	414.1373	437.1266	437.1247	4.3466
*Xylotetraose*	C_20_H_34_O_17_	546.1796	569.1688	569.1651	6.5007
*Xylopentose*	C_25_H_42_O_21_	678.2219	701.2111	701.2090	2.9948

**Table 2:** Composition of mono- and oligosaccharides of different extracts of vine shoots (VS) expressed as g/100g of dry matter.

Compounds	Vine Shoots (VS)		
	H_2_O	H_2_O-EtOH	H_2_O-Amberlyst
Fructose	3.98 ± 0.12 b	8.51 ± 0.04 a	3.64 ± 0.54 b
Sucrose	1.21 ± 0.03 a	0.36 ± 0.03 b	0.41 ± 0.13 b
Xylobiose	2.67 ± 0.31 b	3.53 ± 0.32 a	3.69 ± 0.04 a
Xylotriose	2.65 ± 0.02 a	2.56 ± 0.00 a	2.32 ± 0.06 b
Xylotetraose	7.30 ± 0.71 c	21.35 ± 0.47 a	9.68 ± 0.78 b
Xylopentose	32.84 ± 1.67 c	45.98 ± 0.85 b	51.02 ± 0.98 a
Raffinose	15.86 ± 0.16 a	4.19 ± 0.57 c	11.01 ± 0.16 b
Stachyose	32.45 ± 1.63 a	12.82 ± 0.18 c	17.03 ± 0.46 b
Total xylo-oligosaccarides	45.46 ± 1.70 c	73.42 ± 1.38 a	66.72 ± 0.36 b
Other compounds	1.03	1.34	1.19

Each value is expressed as mean ± SD of three measurements. Different letters in the same row mean statistical differences at *p* < 0.001 (one-way ANOVA and multiple comparisons by Tukey test).

**Table 3:** Composition of mono- and oligosaccharides of different extracts of grape stalks (GS) expressed as g/100 g of dry matter.

Compounds	Grape Stalks (GS)		
	H_2_O	H_2_O-EtOH	H_2_O-Amberlyst
Fructose	17.77 ± 0.49 b	33.19 ± 0.37 a	17.13 ± 0.44 c
Sucrose	0.22 ± 0.03 b	0.29 ± 0.01 a	0.28 ± 0.01 a
Xylobiose	5.29 ± 0.45 b	15.59 ± 0.36 a	5.90 ± 0.17 b
Xylotriose	1.40 ± 0.06 b	5.14 ± 0.26 a	1.69 ± 0.08 b
Xylotetraose	21.53 ± 1.35 a	9.37 ± 0.64 c	13.04 ± 1.12 b
Xylopentose	39.90 ± 1.17 a	25.65 ± 0.77 b	39.86 ± 0.70 a
Raffinose	5.42 ± 0.27 a	4.19 ± 0.07 b	4.54 ± 0.15 b
Stachyose	6.15 ± 0.35 b	3.69 ± 0.09 c	15.09 ± 0.44 a
Total xylo-oligosaccarides	68.13 ± 1.73 a	55.45 ± 0.83 c	60.49 ± 0.83 b
Other compounds	2.32	2.89	2.47

Each value is expressed as mean ± SD of three measurements. Different letters in the same row mean statistical differences at *p* < 0.001 (one-way ANOVA and multiple comparisons by Tukey test).

**Table 4:** Antioxidant activity (ABTS and DPPH) and total phenol content (TPC) in vine shoots (VS) and grape stalks (GS).

	Vine Shoots (VS)			Grape Stalks (GS)		
Parameters	H_2_O	H_2_O-EtOH	H_2_O-Amberlyst	H_2_O	H_2_O-EtOH	H_2_O-Amberlyst
ABTS (µmol TE/g)	65.67 ± 1.80 a	36.41 ± 0.50 b	23.81 ± 0.32 c	58.35 ± 1.25 c	128.04 ± 1.25 a	73.60 ± 0.79 b
DPPH (µmol TE/g)	250.14 ± 0.56 a	148.17 ± 0.56 b	107.71 ± 4.12 c	132.98 ± 4.19 c	425.66 ± 9.99 a	249.45 ± 2.41 b
TPC (mg GAE/g)	58.03 ± 1.80 a	35.80 ± 0.97 b	28.76 ± 0.52 c	49.99 ± 0.07 c	90.92 ± 1.76 a	56.62 ± 0.26 b

Each value is expressed as mean ± SD of three measurements. Different letters in the same row mean statistical differences at *p* < 0.001 (one-way ANOVA and multiple comparisons by Tukey test.

**Table 5:** Identification of the main polyphenols in the extracts from vine shoots and grape stalks detected by LC-ESI-MS/MS.

Compounds	RT (min)	[M − H]^−^	MS/MS Ion	Molecular Formula	Reference
Citric acid	2.342	191.0197	111.0123; 173.0107; 191.0202	C_6_H_8_O_7_	[55,63]
Gallic acid	6.032	169.0170	125.0278	C_7_H_6_O_5_	[52,53,54,56,57,59,61,62,63,64]
Monogalloyl glucose	8.417	331.0669	313.0569; 169.0167; 168.0093; 125.0293	C_13_H_16_O_10_	[53,57,60,62,64]
Caftaric acid	9.002	311.0409	111.0113; 173.0120	C_13_H_12_O_9_	[52,54,56,58,59,61,63,64]
Coutaric acid	9.407	295.0453	112.9935; 149.0132; 163.0425; 251.0447	C_13_H_12_O_8_	[53,56,58,60,61,63]
Quercetin-3-*O*-glucuronide	11.150	477.0702	175.0280; 301.0357; 413.0928	C_21_H_18_O_13_	[56,58,60,61,63]
Kaempferol-3-*O*-glucoside	11.842	447.0907	150.9993; 255.032; 285.0396; 32.0492	C_20_H_20_O_11_	[58,59,61]
Taxifolin	12.787	303.0481	125.0428; 177.019; 285.0536	C_15_H_12_O_7_	[56,58,59,61]

## Data Availability

The data are available on request.

## Sample Availability

Samples of the compounds are available from the authors.
